# Domestication and large animal interactions: Skeletal trauma in northern Vietnam during the hunter-gatherer Da But period

**DOI:** 10.1371/journal.pone.0218777

**Published:** 2019-09-04

**Authors:** Rachel M. Scott, Hallie R. Buckley, Kate Domett, Monica Tromp, Hiep Hoang Trinh, Anna Willis, Hirofumi Matsumura, Marc F. Oxenham

**Affiliations:** 1 Department of Anatomy, University of Otago, Dunedin, New Zealand; 2 College of Medicine and Dentistry, James Cook University, Townsville, Australia; 3 Department of Archaeology, Max Planck Institute for the Science of Human History, Jena, Germany; 4 Department of Prehistoric Archaeology, Vietnam Institute of Archaeology, Hanoi, Vietnam; 5 College of Arts, Society and Education, James Cook University, Townsville, Australia; 6 School of Health Science, Sapporo Medical University, Sapporo, Hokkaido, Japan; 7 School of Archaeology and Anthropology, Australian National University, Canberra, Australia; Ohio State University, UNITED STATES

## Abstract

The aim of this paper is to test the hypothesis that healed traumatic injuries in the pre-Neolithic assemblage of Con Co Ngua, northern Vietnam (*c*. 6800–6200 cal BP) are consistent with large wild animal interactions prior to their domestication. The core sample included 110 adult (aged ≥ 18 years) individuals, while comparisons are made with an additional six skeletal series from Neolithic through to Iron Age Vietnam, Thailand, and Mongolia. All post cranial skeletal elements were assessed for signs of healed trauma and identified cases were further x-rayed. Crude trauma prevalence (14/110, 12.7%) was not significantly different between males (8/52) and females (5/37) (χ^2^ = 0.061, *p* = 0.805). Nor were there significant differences in the prevalence of fractured limbs, although males displayed greater rates of lower limb bone trauma than females. Further, distinct from females, half the injured males suffered vertebral fractures, consistent with high-energy trauma. The first hypothesis is supported, while some support for the sexual divisions of labour was found. The prevalence and pattern of fractured limbs at CCN when compared with other Southeast and East Asian sites is most similar to the agropastoral site of Lamadong, China. The potential for skeletal trauma to assess animal trapping and herding practices prior to domestication in the past is discussed.

## Introduction

The use of animals for their meat, milk, and labour had an enormous influence on past population structure and health. With the emergence and intensification of agriculture, the cultivation of crops increased productivity and communities grew with increased food outputs. Changes in foodstuffs affected protein and vitamin availability and intakes, markedly influencing physiological health [1]. Equally, interacting with large animals during hunting, corralling, managing wild herds, and eventual domestication must have exposed individuals to the risk of minor through to serious injury, including the associated risks of infection and debilitating co-morbidities [2, 3].

The analysis of skeletal trauma in the past can highlight the physical risks faced by individuals within a community navigating the transition from hunting and gathering to using and consuming domesticated products, including animals. Few studies have investigated the physical risks of wild animal interactions in the past and while research has been conducted on skeletal trauma in Southeast Asia [4–7], the current research specifically explores such trauma in a pre-farming group from the region. This study investigates the frequency, pattern, and types, of post cranial skeletal trauma in a group of individuals who lived during the Da But period in northern Vietnam 6800–5500 BP. This period predated the emergence of farming by at least 2500 years and is characterized by a subsistence base that included large wild animals and a marked increase in the number of large, potentially sedentary, hunter-gatherer communities in the region [6, 8–11]. The primary aim of this paper is to characterize the trauma patterns of the Da But period pre-farming settlement of Con Co Ngua (c. 6800–6200 cal BP.).

### The biocultural context of Con Co Ngua

**Fig 1:**
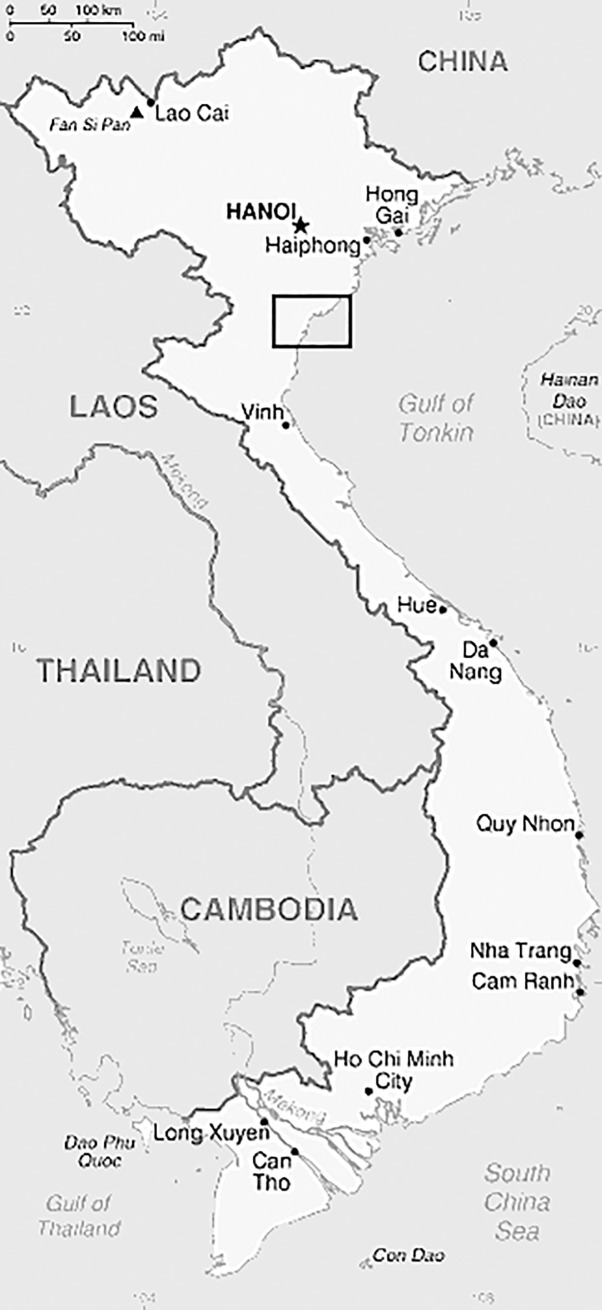
Box insert is the region of the Da But period site of Con Co Ngua, northern Vietnam. (Map sourced from https://www.cia.gov/library/publications/the-world-factbook/attachments/maps/VM-ma).

Archaeological excavations were first conducted at Con Co Ngua (c. 6800–6200 cal BP) by the Institute of Archaeology, Hanoi in 1979/80 [8, 10]. Con Co Ngua (CCN) is situated in a small valley approximately 3km north, 4km east of the Ma River in the Bac Bo plain, Thanh Hoa province, 30 km from the present coast of the Gulf of Tonkin, South China Sea [12] (Fig 1). Formerly a raised mound, it is comprised of midden, pottery, lithics, and stone artefacts in addition to a high density of human burials. The first excavations recovered over ninety individuals including adults and children buried in both squatting (within pits) and side-flexed positions, some of which exhibited signs of serious healed trauma [6, 8, 9, 11]. In addition to falls, interacting with large animals (such as water buffalo) through hunting or corralling was implicated as one potential factor responsible for the observed frequency and pattern of healed skeletal trauma [6, 10]. In 2013, further excavations at CCN led by MFO and HHT recovered additional burials. As with the first excavations, individuals were buried squatting or side flexed with evidence of systematic postmortem mutilation, including chopping of long bones, clavicles, occasional head removal of the corpse, and subsequent tight wrapping (likely with bark cloth) of the body [10].

The natural environment and wildlife in northern Vietnam is diverse with potential for a range of accidental trauma to occur. Botanical and zoological remains excavated from the site, situated within a low-lying valley and surrounded by hills up to 350m high, derive from freshwater and coastal areas including rocky reefs, mangroves, alluvial grasslands, riparian forests, and woodlands [10, 12]. Traversing these environs would have exposed individuals to the risk of tripping and falling injuries. Further, typhoons, storm surges, flooding, landslides, and strong winds known to occur in Vietnam could have increased the chances of accidental injuries [13, 14]. Indirectly, these weather events could have disrupted the food web, forcing inhabitants to become more mobile and travel over wider areas in search of food further exposing them to risk. No weaponry was found at the site, although bevelled axes, large hammer stones, and grindstones were recovered that could have caused trauma [10], in addition to opportunistic weaponry such as sticks (and fists) not visible in the archaeological record [15, 16].

Modes of subsistence influence physiological health indirectly by encouraging vector borne diseases through animal husbandry and irrigation practices, and zoonotic pathogen transmission [2, 17]. Subsistence practices also directly expose humans to physical risks. Midden material covering the burials provides some evidence for the subsistence base of the community buried at CCN. The botanical remains of fruits and nuts, skeletal remains of mammals, reptiles, birds, and aquatic species were present [10]. Vertebrate remains (none of which are domesticated and many of which show signs of butchering and processing) include pig, a tiger mandible, deer, arboreal primates, with water buffalo (*Babalus bubalis*) dominating the assemblage [10, 12]. The frequency of bovid remains arguably increased over time suggesting a preference for their meat, better hunting strategies, or management of the wild herds (Bui Vinh, 1996 cited in Oxenham, 2006) [12]. The age structure of the bovids indicates adults were targeted, suggesting juveniles may have been spared to maintain wild herd numbers; in populations of large wild herbivores there is a higher proportion of live juveniles left in groups harvested by humans [18]. This form of selective harvesting to propagate herd numbers has been hypothesised for other archaeological assemblages [2]. The presence of a calcified cyst associated with one burial, and skeletal lesions possibly caused by hydatid disease, further support a close relationship between humans and ungulates at the site [10].

Bovids can easily cause injury to humans when provoked, fearful or excited, when protecting calves, or accidentally during handling [19, 20]. Published research of the human injuries caused by bovids note the common mechanisms of trauma as being tossed, crushed, kicked, trampled, head-butted, or gored [21–25].

In this study, the types and prevalence of antemortem post cranial skeletal trauma in the adult sample excavated in 2013 are analysed to readdress the potential causes for trauma with a focus on the influence of subsistence practices and risks to humans during the Da But period. Due to the absence of traditional weaponry recovered from the site and the ostensible management and hunting of wild buffalo herds, it is hypothesised that injuries consistent with large wild animal interactions will be expected in higher frequencies compared with injuries more commonly associated with accidents such as falls and trips. We further hypothesise that injuries will be more frequent in males compared with females if traditional assumptions concerning the sexual division of labour operated during this period. We do, however, recognise that there is archaeological and contemporary evidence that females regularly participate in hunting activities [26]. Globally, few studies examine the potential risk of trauma with respect to hunting methods, large vertebrate management practices, and potential emergent pastoralism in the past. With the additional skeletons excavated from CCN in 2013, we have the opportunity to broaden our investigation into the interplay between health and ostensibly intensive wild animal interaction in this hunter-gatherer community in the diverse subtropical environment of northern Vietnam.

## Materials and methods

One hundred and seventy-two individuals were recovered in 2013 comprising 62 subadults aged <18 years and 110 adults. Subadults are not included in the analysis as different risk factors may have impacted younger age categories [27]. The skeletal material is curated at the Institute of Archaeology, Hanoi.

Adult sex was estimated using a combination of non-metric pelvic [28, 29] and cranial [30] morphology in addition to CCN-specific metric sex estimation functions developed on the 1979/80 sample [8]. As noted, age-at-death, for the purposes of this paper, was restricted to the adult (18+ years old) sample and was based on dental wear seriation (see [31]. Maxillary and mandibular first and second molar wear scores [32] were recorded for each individual. Missing data were filled with available and relevant antimere tooth wear data. In cases of postmortem tooth loss, the maximum tooth wear score (maximum score of 40) was entered for individuals where there was evidence for advanced tooth wear (where scores of 30+ for preserved teeth occurred). The entire adult sample was then seriated based on average mandibular M_1_+M_2_ tooth wear. The remaining un-seriated individuals (those that could not be seriated based on averaged mandibular M_1_ and M_2_ scores) were then added to the seriation based on where their remaining tooth scores fell. The sample was then arbitrarily divided into three age classes: young adults (average wear score less than 19.9), middle adult (wear score 20 to 29.0), old adult (wear score 30+) and un-seriated adults (no wear information available). As a check on the general validity of the seriation, individuals for whom independent age-at-death estimates could be made (e.g. pubic symphyseal age, epiphyseal fusion) were identified in the seriation. There were no instances of young, middle or old adults, based on independent age-at-death estimates, erroneously falling into unexpected age classes in the seriation. Due to poor preservation of skeletal elements usually used in age estimation, the method of dental wear seriation enabled a larger proportion of the assemblage to be arranged within age categories. Traditionally, these adult age categories are placed with the age ranges of young (20–29), mid (30–49), and old (50+) [33]. However, it is unclear how these actually correlate to age categories in the past and therefore young, mid, and old are used as an assemblage specific description of the relative age of adult individuals.

The age and sex distribution of the adult individuals are summarised in Table 1. While there are more males than females (1.4:1) the proportion is not significantly different from expected (binomial test *p* = 0.137, two tail). Moreover, the frequency of young, middle and old adults is not statistically significantly different from each other (χ^2^ = 3.928, p = 0.140). While fragmented, bone preservation was very good to excellent allowing detailed recording of the skeletal pathology.

**Table 1:** Age and sex distribution of Con Co Ngua adults.

	Male	Female	Unsexed	Total	%
Young	19	13	7	39	35.5
Mid	17	11	4	32	29.1
Old	15	10	1	26	23.6
Adult	1	3	9	13	11.8
Total	52	37	21	110	
%	47.3	33.6	19.1		

Complete and fragmented skeletal elements were macroscopically examined for evidence of antemortem skeletal trauma. Due to the practice of occasional head removal, cranial trauma had to be excluded from the analysis. Analyses were carried out prior to the reconstruction of any elements. In some cases, however, elements were reconstructed in order to assess for angulation and apposition of healed fractures. Antemortem trauma was classified based on evidence of osseous remodelling at the site of a defect indicating that healing had begun during the individual’s life. Potential perimortem trauma (at or around the time of death) was excluded from analysis because the complex mortuary treatment at CCN involved deliberate modification of skeletal elements after death. Perimortem injuries to bone do not show evidence of remodelling and breaks are characteristic of ‘fresh’ bone that can include distinctive fracture angles, evidence of bone peeling, and colour [34, 35]. A caveat to this is that evidence of bone remodelling (healing) may not be visible if death occurs within approximately one week of injury. Thus, potential non-survivors of traumatic events are not represented here. Only skeletal elements with visually evident signs of healed trauma were radiographed. Where radiographs revealed an underlying non-traumatic pathological process, the affected elements were excluded from further analysis. Fracture prevalence was calculated for limb bones, including clavicles, based on the number of elements in the sample ≥75 percent complete [33, 36]. Therefore, frequency data for each affected element differs. Incomplete elements (<75 percent complete) were included in the analysis if they exhibited signs of trauma. This method is preferred over using the number of individuals as the denominator, which does not take into account missing elements and it therefore enables more direct comparisons with other skeletal samples [15, 37].

The degree of angulation of limb bone fractures was used as a barometer for the success of healing and one indicator that treatment may have been provided for individuals. Bamboo is a valuable resource now and in antiquity, and in addition to its many medicinal qualities, it is used to make splints for the aid of fracture repair [38, 39]. In various regions of Asia other flora (for example *Drymoglossum heterophyllum*) are used traditionally to make a poultice that is applied to fractured bones or dislocations [40]. Angular deformity can cause functional impairment and has implications for the ability to perform daily activities so is important to identify with respect to possible functional costs of a traumatic event. Fracture angles were measured from radiographs using a goniometer aligned parallel and vertically with the centre of the medullary cavity of each fragment of the injured limb bone following Grauer and Roberts [41] and Roberts [42].

The prevalence of trauma between males and females, by age class (young, middle and old adult), between upper and lower limbs, and between comparable sites in Southeast and East Asia were tested for statistical significance using Chi Square (χ^2^), the most appropriate approach when comparing frequency data. Alternatively, and where fewer than five observations occurred, Fishers Exact Test (FET) was used (two-sided) [43]. The significance threshold was set at *p* < 0.05. Statistical analyses were conducted using IBM SPSS Statistics Version 24 and online calculators in some instances (http://statpages.info).

### Skeletal trauma analysis

Bone is a dynamic tissue with the ability to remodel and as such, in the case of healed trauma, it is most often impossible to estimate at what age an individual suffered an injury. In addition, when the skeleton of an individual exhibits multiple healed traumas it is extremely difficult, if not impossible, to ascertain if the injuries were sustained in one traumatic event or over the person’s lifetime [44]. In human skeletal remains, the bones fractured, distribution of injuries, types of fracture lines, and associated cultural context can help to interpret the mechanism of injury in the past [34].

Skeletal fractures occur when external forces acting on the body push the organic structure of muscle and bone beyond their mechanical capabilities resulting in the deformation or fracture of soft and hard tissues. When forces are placed on bone pushing it past the stress/strain threshold it will reach breaking point [45]. Forces include compression, tension, torsion, bending, or any combination of these [45, 46]. Different forces and mechanisms of injury result in different fracture types and fracture lines. Fracture lines and the fracture morphology of long bones are categorized as transverse fractures (bending forces), oblique fractures (compression, bending, and torsion), spiral fractures (torsional load), butterfly fractures (bending and compression forces), impacted fractures (compression forces), comminuted fractures (at least three fracture fragments), and greenstick fractures (bending load causing an incomplete fracture) [34, 47]. By identifying the fracture line, the type of force responsible can be inferred. Transverse, comminuted, and crushing fractures are more often caused by direct trauma, whereas oblique and spiral fractures are more often the result of indirect trauma [34]. Both direct and indirect forces can result in blunt trauma causing fractures. Sharp force trauma occurs when a foreign object penetrates or strikes the body, for example a projectile or bladed weapon [48, 49]. Sharp force injuries can be identified on bone by a v- or u-shaped impression, striations, angled impressions, and smooth walls [49–51].

### Comparative assemblages

The adaptation of subsistence practices in Southeast and East Asia has been investigated archaeologically including the analysis at some sites of antemortem limb bone fractures. The results from these studies compared with the Con Co Ngua data may help to elucidate the patterns of trauma experienced by the people buried at CCN and in the greater region during periods of subsistence and cultural change.

Metal period sites (3300–1700 BP) in northern Vietnam are provided as a direct regional comparison to the CCN series. Following the Da But period, there is evidence for farming, the exploitation of domesticated plants (particularly rice) and animals (especially pigs and dogs) in the region from at least 4000 BP, while some degree of population intensification is seen with the emergence of the Bronze and Iron Ages (3300–1700 BP) [6, 9]. Three Thai and two Chinese sites are included for a broader regional comparison.

The Thai sites consist of the Neolithic coastal site of Khok Phanom Di (n = 68) (3950–3450 BP), Southeast Thailand, the inland Bronze Age site of Ban Lum Khao (n = 59) (3350–2450 BP), Northeast Thailand [4], and the inland site of Non Ban Jak (n = 55) (1650–1200 BP), Northeast Thailand [7]. The site of Khok Phanom Di is represented by foodstuffs of a marine and plant base, including rice, but there is no evidence for animal domestication [52, 53]. The Bronze Age site of Ban Lum Khao had an increased reliance on rice agriculture and animal husbandry, which potentially exposed them to different risk factors [4]. Non Ban Jak bridges the late Iron Age and protohistoric periods, during which production of wet rice agriculture intensified with evidence for domesticated cattle and water buffalo [54]. Other Thai sites where fracture rates have been calculated (for example Ban Na Di and Nong Nor) were excluded as comparative examples due to their small sample sizes and/or relatively poor preservation [4].

Further north, the Chinese samples include the pastoral site of Jinggouzi, Inner Mongolia (n = 64) (2950–2550 BP) and the agropastoral site of Lamadong, Northeast China (n = 443) (1730–1511 BP) [55]. Twenty-eight tombs were excavated at Jinggouzi, twenty-five of which contained the bones of animal domesticates. The tombs of these pastoralists also included weaponry and artefacts related to animal husbandry and horseback riding [55]. At the site of Lamadong, inhabitants practiced a mixed economy. Tombs at the site included artefacts such as weapons, helmets, horseback riding paraphernalia, and ploughs and sickles suggesting agriculture was established at that time [55].

## Results

### Rates of trauma

There was no evidence of healed antemortem sharp force trauma among any adult individuals. A summary of trauma by individual is provided in Table 2. The crude prevalence of trauma by individual, not accounting for differential preservation, was 12.7% (14/110) affecting 14.4% (8/52) males, 13.5% (5/37) females, and 4.8% (1/21) adults of indeterminate sex. Neither sex fractured their scapulae (males 0/74, females 0/53) or sternum, although preservation of the latter was extremely poor (males 0/8, females 0/1), which is not unusual for archaeological groups. There was no significant difference between the number of males and females with a traumatic bone injury (χ^2^ = 0.061, *p* = 0.805).

**Table 2:** Summary of antemortem (healed) fractures in individuals from Con Co Ngua (2013 excavation).

Burial	Adult age	Sex	Element	Description of injuries
M2a	Mid	Male	Clavicle	R clavicle: mid shaft fracture. The acromial end is bowed superiorly and there is a deep lytic defect on the inferior aspect
			Radius	R radius: colles’ fracture of the distal metaphysis. R ulna not injured
			Ribs	Ribs (3) un-sided: shaft fractures
M9a	Mid	Female	Clavicle	L clavicle: well remodeled fracture just medial to the muscle attachment of the deltoid on the acromial extremity. Orientation is ant/posterior. No displacement
M44a	Young	Male	Lumbar vertebrae	L1: compression fracture. Anterior height 18.7mm, posterior height 25.5mm
				L2: fractures of the right pedicle and left lamina
M48a	Young	Female	Radius	R radius: comminuted fracture of the distal diaphysis. Distal portion is displaced laterally and bowed anteriorally, 17° angulation.
				Distal R ulna not present
M69a	Mid	Female	Ulna	L ulna: oblique fracture of the distal diaphysis. Lateral displacement of distal portion, 18° angulation. L radius not present
M77a	Old	Male	Lumbar vertebrae	L1: left pedicle fractured and shortened with associated osteophyte formation. Left inferior facet is enlarged with osteophyte formation and eburnation
				L2: corresponding superior facet is similarly affected as L1
M89a	Unknown	Unknown	Metacarpal	L mt5: Jones’ fracture of the proximal shaft
M114a	Mid	Male	Radius	L radius: possible Chauffer’s fracture of the styloid process. Slight anterior displacement. L ulna (distal third) not present
M117a	Young	Male	Radius	L radius: oblique fracture of the proximal diaphysis, 8–10° angulation. Left ulna not injured
			Ulna	R ulna: transverse fracture of the mid shaft, 10° angulation. R radius not injured
			Tibia	L tibia: comminuted fracture of the distal diaphysis. Slight lateral displacement, 3–8° angulation. Callus approximately 10cm long still remodelling. Cloaca on distal posterior shaft
			Fibula	L fibula: oblique fracture of the distal diaphysis, 18–25° angulation. Possible supination external rotation fracture (supinated foot)
M124a	Old	Male	Phalanx	R hand phalanx: fracture third digit distal and intermediate phalanges fused
M126	Mid	Male	Femur	L femur: transverse fracture of the mid shaft. The callus does not encompass the anterior portion of the fracture and is well remodelled, 8–16° angulation
			Pelvis	L pubic ramus: fracture on the ventro superior aspect just adjacent to the acetabulum. The rami of the pubis are significantly shorter than the right side
			Lumbar vertebra	L2: compression fracture
M127a	Mid	Male	Clavicle	R clavicle: fracture of the acromial end with slight displacement medially and slight inferior rotation
			Cervical vertebrae	C3 or C4: compression flexion or vertical compression fracture
M133a	Mid	Female	Metatarsals	L mt4 and mt5: non union/amputation
M140	Old	Female	Radius	R radius: oblique fracture of the distal diaphysis, 13–15° angulation. Slight posterior displacement. R ulna not injured
			Ulna	L ulna: fracture of the distal diaphysis. Distal shaft pushed upwards and medially causing overlap and displacement, 3–8° angulation. Partial left radius present and not injured

**Fig 2:**
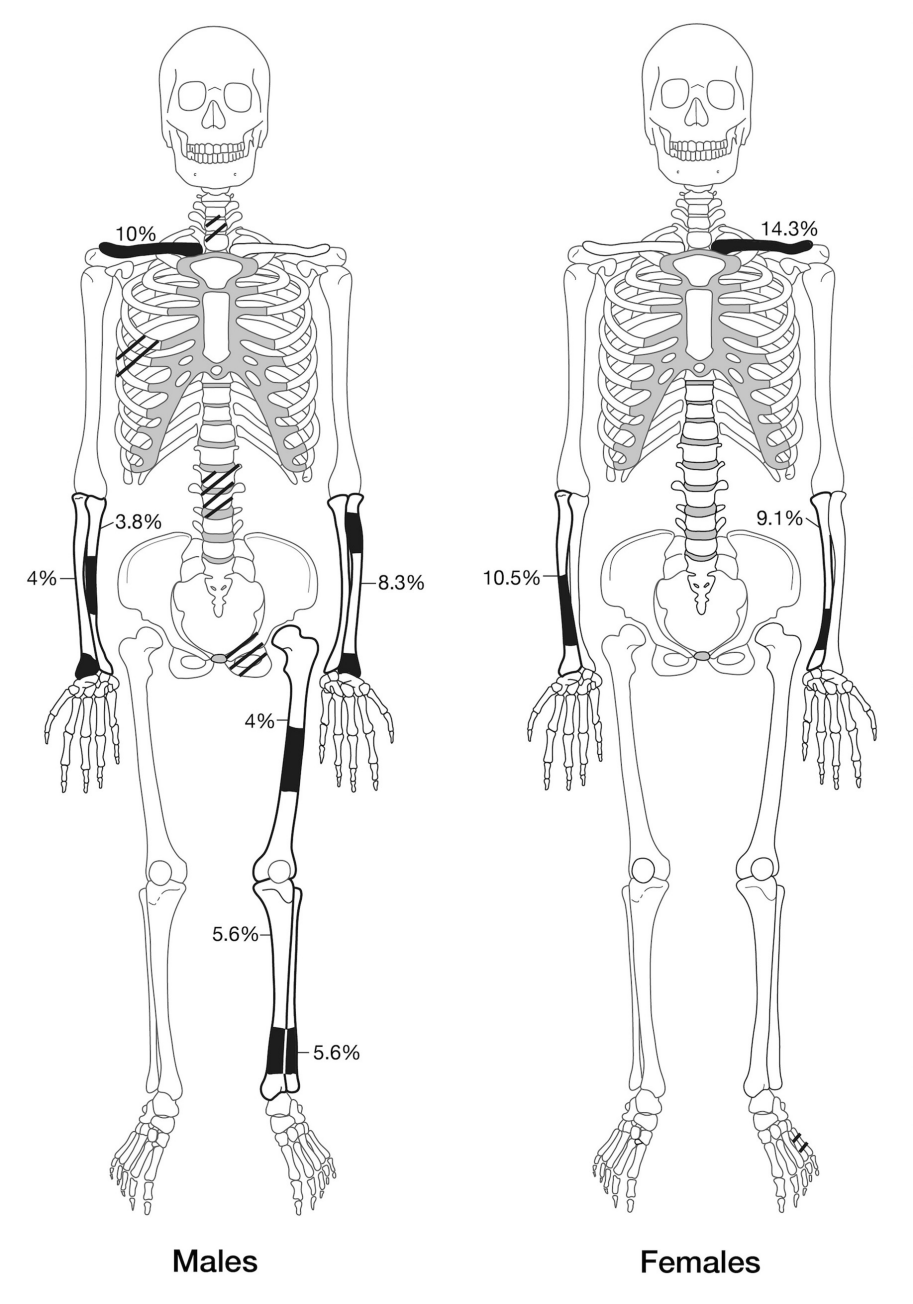
Regions of the skeleton with antemortem trauma of males and females. Areas not quantified by the number of observable elements are indicated by striped regions.

The prevalence of fractures by the number of observable limb bones (including clavicles) ≥75 percent complete is provided in Table 3 (see also Fig 2). When the total number of observed limb bones are considered there were no significant differences in fracture frequencies between the sexes (χ^2^ = 0.050, *p* = 0.823). Also, neither sex had fractured individual limb bones significantly more than the other. No age category suffered significantly (χ^2^ = 2.639, p = 0.267) more trauma (young 3/35, 8.6%; mid 7/32, 21.9%; old 3/26, 11.5%). Males displayed similar fracture rates (FET = 0.530) between the upper (6/198, 3.0%: clavicle, humerus, radius, ulna) and lower (3/122, 2.5%: femur, tibia, fibula) body. Females had higher, albeit not statistically so (FET = 0.124), upper body (5/133, 3.8%) fracture rates in comparison to the lower body (0/68, 0%). Male and female upper body fracture rates were similar (χ^2^ = 0.485, p = 0.486), while male lower body fractures rates were higher, although not statistically so (FET = 0.262). In addition to these trends, males also displayed more rib fractures, and fractures to their lumbar and cervical vertebrae (Fig 2). Limb bone fractures of both sexes had healed successfully with minor (<10°) to moderate angulation of the diaphyses [56]. No elements exhibited angulation to a degree considered to constitute ‘unsuccessful healing’ [41, 42].

**Table 3:** The prevalence of limb bone fractures (including clavicles) at Con Co Ngua by side and sex.

Element	Males		Females		Significance*	Indeterminate		Total	
	n/N (L + R = total)	%	n/N (L + R = total)	%		n/N (L + R = total)	%	n/N (L + R = total)	%
Clavicle	0/17 + 2/20 = 2/37	5.4	1/7 + 0/16 = 1/23	4.3	p = 0.675*	0/5 + 0/4 = 0/9	0	1/29 + 2/40 = 3/69	4.3
Humerus	0/22 + 0/33 = 0/55	0	0/18 + 0/20 = 0/38	0	N/A	0/7 + 0/2 = 0/9	0	0/47 + 0/55 = 0/102	0
Radius	2/24 + 1/25 = 3/49	6.1	0/12 + 2/19 = 2/31	6.5	p = 0.647*	0/4 + 0/5 = 0/9	0	2/40 + 3/49 = 5/89	5.6
Ulna	0/31 + 1/26 = 1/57	1.8	2/22 + 0/19 = 2/41	4.9	p = 0.377*	0/6 + 0/6 = 0/12	0	2/59 + 1/51 = 3/110	2.7
Femur	1/25 + 0/26 = 1/51	2.0	0/17 + 0/15 = 0/32	0	p = 0.614	0/6 + 0/6 = 0/12	0	1/48 + 0/47 = 1/95	1.1
Tibia	1/18 + 0/16 = 1/34	2.9	0/8 + 0/10 = 0/17	0	p = 0.667	0/4 + 0/4 = 0/8	0	1/30 + 0/30 = 1/60	1.7
Fibula	1/18 + 0/19 = 1/37	2.7	0/10 + 0/9 = 0/19	0	p = 0.661	0/4 + 0/2 = 0/6	0	1/32 + 0/30 = 1/62	1.6
Total	5/155 + 4/165 = 9/320	2.8	3/93 + 2/108 = 5/201	2.5	χ^2^ 0.050, p = .823	0/36 + 0/29 = 0/65	0	8/285 + 6/302 = 14/587	2.4

n is number of antemortem fractures; N is the observed number of elements >75% complete (left + right)

* Fisher Exact Test (FET), males compared with females

Over half of the individuals with skeletal trauma had multiple injuries (8/14, 57.1%). More males (4/8) than females (1/5) with trauma had fractured one limb bone in addition to at least one other element, although the difference was not statistically significant (FET p = 0.315). This includes one male and one female who had fractured more than one limb bone each. The male (M117a) had suffered a fractured left radius, right ulna, left tibia, and left fibula, while the female (M140a) had fractured her right radius and left ulna. Limb bone fractures in both sexes are a combination of distal metaphysis, epiphysis, and oblique and transverse fractures suggesting indirect and direct forces.

### Trauma case studies

In this section the various healed fractures listed in Table 2 are described in order to explore the proximate (mechanism) causes of trauma which, in turn, allows informed inferences regarding the ultimate (or behaviourally mediated) causes of trauma. However, only limited discussion of ultimate causes is dealt with here, with a fuller review of potential behaviours relating to these instances of trauma dealt with in the discussion section. As noted, only instances of healed or healing trauma are explored. No cases of non-union and/or trauma in the early stages (first week) were noted. Due to difficulties in precisely determining time since original trauma (e.g. see Lovell 1997), no attempt is made to estimate time since trauma in the following cases. Notwithstanding, the degree of callus formation and remodelling seen in each of the described cases suggests a minimum period of three to six months (and potentially more often likely years) since fracture initiation (Nork, 2006 cited in [57].

### Limb bone fractures (see Table 2)

A middle aged adult male (M2a) had a well-healed Colles’ fracture of his right radius with no visible fracture line. Today, Colles’ fractures are common injuries and can occur from a standing fall onto an outstretched hand [58]. This individual also had a mid-shaft fracture of their right clavicle, and three rib fractures to the mid-thoracic region. Like Colles’ fractures, clavicle fractures are common today, and often occur concomitantly with rib fractures, head and neck trauma, and upper extremity injuries [59, 60]. Clavicle fractures are caused by medium to high-energy trauma either accidentally from a fall to the ground, from an accidental or intentional force (e.g. a direct blow), or from laterally directed force to the shoulder [59]. A mid aged adult female (M9a) and mid aged adult male (M127a) had also fractured a clavicle. One adult male (M114a) had suffered an intra-articular fracture of the radial styloid process of his left radius (Chauffeur’s/Hutchinson’s fracture). These occur from direct force to the wrist or tension, such as when the hand is forcibly bent backwards (dorsiflexion) and away from the body (abducted) [61].

A young adult female (M48a) fractured the diaphysis of the distal third of her right radius. Radiographs suggest this may have been a comminuted fracture caused through high-energy forces. Her distal right ulna was not present. An old aged adult female (M69a) had fractured the distal diaphysis of her left ulna. The fracture line is oblique, which is more typical of indirect force, which may suggest accidental injury. Behaviours associated with such trauma could include a fall from a height or a low-energy fall onto an outstretched hand [62]. If the radius is not involved then direct force to the diaphysis could also have been responsible [62]. Unfortunately, her left radius was not present. However, the left ulna is less often fractured from direct force compared with the right as individuals generally raise their dominant arm to ward of a blow (parry fracture). Finally, an adult female (M140a) had healed fractures to the mid shaft of her right radius and distal half of the diaphysis of her left ulna. Her right ulna was present and had no evidence of injury. The fracture line of the right radius is oblique, indicating indirect force, which is more consistent with an accidental aetiology. The fracture line of the fractured left ulna was unable to be determined and the left radius was fragmentary.

Half of those individuals with antemortem trauma at CCN had multiple injures which can occur from high energy events. These are less likely to be accidental in nature and require further consideration.

### Case study: Multiple traumas M117a

**Fig 3:**
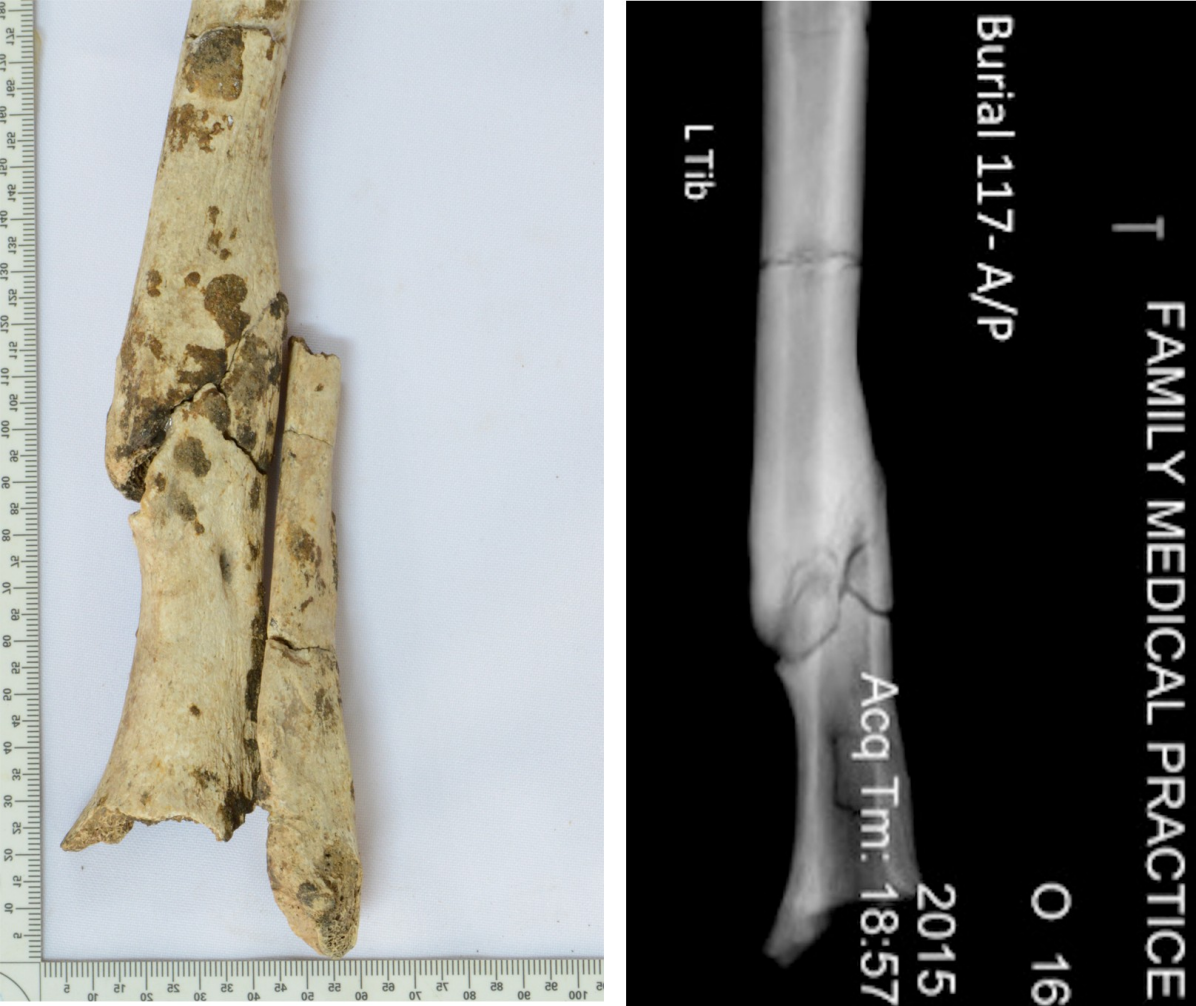
Left tibia and fibula (distal half of diaphysis, anterior view), and corresponding radiograph (tibia only, anterior view) of a young adult male buried at Con Co Ngua (Burial M117a). Note oblique fracture angle (in radiograph) and residual callus formation on tibial diaphysis].

A young adult male (M117a) experienced multiple fractures to his upper (left radius and right ulna) and lower limbs (left tibia and left fibula). All adjacent elements were present and were not injured. The oblique fracture (indicating indirect force) of the radial diaphysis suggests an accidental mechanism. The transverse fracture of the mid diaphysis of his ulna suggests direct force trauma. The fracture of the distal third of the left tibia has a large callus approximately 10 cm in length that was in the process of remodelling at the time of his death, with a cloaca on the posterior shaft, suggesting secondary bone infection (Fig 3). Tibial fractures are common today and often involve soft-tissue trauma [63, 64]. Although they can be treated without surgery they are often debilitating [63, 64]. Radiographs show a comminuted fracture with butterfly fragment, a type of fracture which occurs during high-energy trauma from a direct force or fall from a height [64]. The fibula has an oblique fracture at the distal diaphysis/ankle. Ankle fractures are often caused by indirect trauma [65] from standing falls or falls from a height [66]. The oblique fracture line and location of the fracture suggests the trauma was due to bending forces, possibly a supination external rotation fracture (supinated foot/rolled ankle) [66]. It is not possible to determine if these fractures occurred in a single event or during multiple events.

### Case study: Multiple traumas M126a

A mid aged adult male (M126a) had suffered multiple fractures including his left femur, left pubic ramus, and a compression fracture of the second lumbar vertebra. The left femur was fractured at the mid-diaphysis. Femoral and pubic ramus fractures are caused from high-energy trauma [67–69]. Concomitant injuries to femoral fractures can include spinal compression fractures (Ricci, 2008), acetabular fractures, and pelvic ring injuries [68]. Concomitant injuries to the pubic rami trauma can include injuries to the chest, long bones, and spinal fractures [67]. Both femoral and pelvis fractures can cause disability and chronic pain [69, 70]. Fractures to the pubic ramus can occur during falls, equestrian accidents, as crush injuries [67], and by bovine attacks [23, 71].

### Vertebral fractures

**Fig 4:**
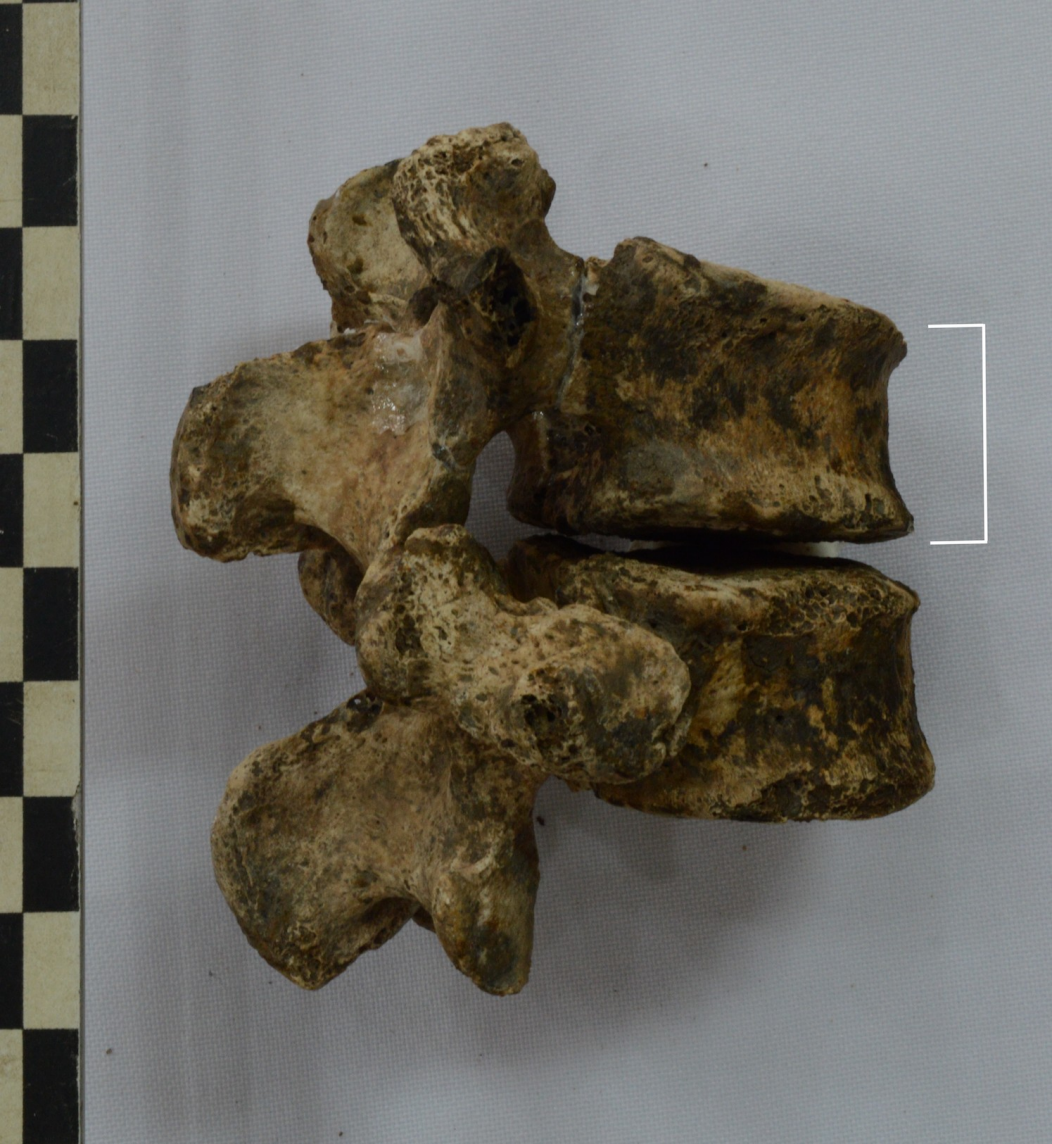
Compression fracture of the first lumbar vertebra (upper body in the photo). Right postero-lateral view, young adult male (M44a).

Four adult males had vertebral injuries (M44a, M77a, M126a, M127a). Three of these had injured lumbar vertebrae and one mid aged male (M127a) had a vertical compression fracture of his subaxial cervical spine (below C2), which occurs from compressive flexion or vertical compression forces, for example a combination of hyperflexion and compression [72, 73]. The other individuals had fractured one or both of their first and second lumbar vertebrae including compression fractures and fractured lamina and pedicles (Fig 4).

### Non-union and amputation

**Fig 5:**
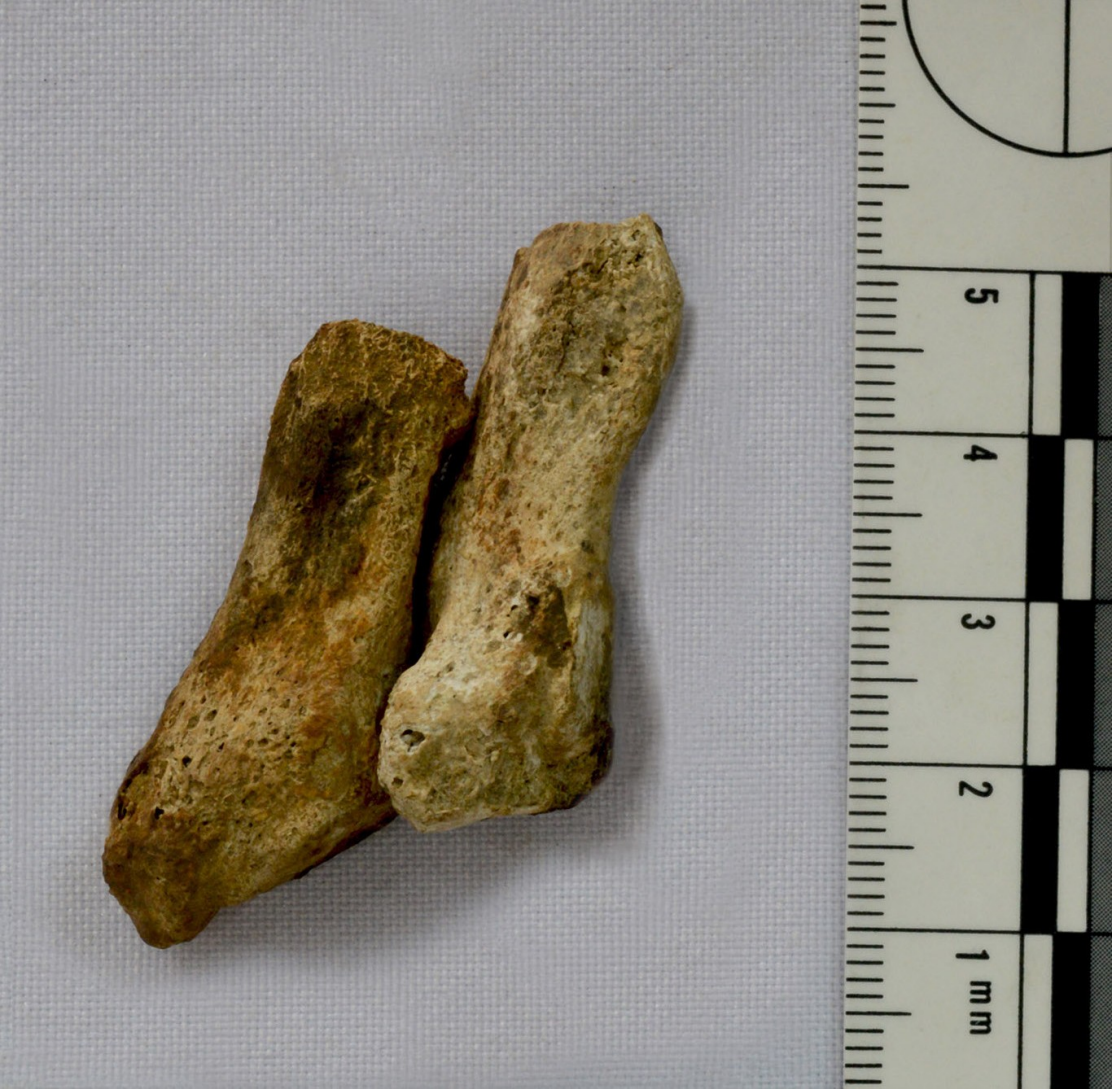
Left metatarsals four and five (dorsal aspect) of a mid-aged adult female buried at Con Co Ngua (Burial M133a). The distal ends (originally mid-shafts) display equivalent levels of extensive remodelling (but lack bridging callus formations) consistent with non-union and/or amputation.

A mid aged female (M133a) had lost the distal half of her fourth and fifth left metatarsals (Fig 5). The ankle joint of this individual has signs of degenerative joint disease and other severe arthritic changes including complete ankylosis of the talo-navicular joint, which may be related to limited mobilization and mechanical stress placed on the foot following an injury. The diaphysis of the fifth metatarsal shows some signs of atrophy and the preserved distal end has been partially resorbed. The lack of callus formation on the margins of the preserved distal ends of the fourth and fifth metatarsal diaphyses indicates that non-union occurred. The lack of any callus formation, in the context of extensive remodelling at the site of trauma, is consistent with hypovascularity inhibiting the formation of a bridging callus [74, 75]. Clinically, avascular non-unions will not unite even with immobilization of the region [74, 75]. It is possible that the distal fragments of the metatarsals were severed at the time of a traumatic incident, or later through infection and necrosis [74]. Open metatarsal fractures more commonly occur from a direct blow or crushing [76]. It is also possible that non-union occurred following complete stress fractures of Mt4 and Mt5 although this is less likely as both bones are involved [77]. Incidentally, other foot and hand trauma in the sample included M89a (sex unknown) who showed evidence for a healed fifth metatarsal fracture (Jone’s fracture) and M126a (male) whose right third distal and intermediate phalanges had ankylosed.

### Comparisons with other Southeast Asian assemblages

Here we compare the frequency of pattern of trauma at Con Co Ngua with other archaeological assemblages in Southeast Asia. The prevalence rates of limb bone fractures from the 1979 excavation of CCN [6] were combined with the current data and are presented in Table 4 with comparable sites in Southeast and East Asia (where limb bone frequencies were available). While the total frequency (excluding clavicle fractures) ranges from 0.0% (Khok Phanom Di) to 3.5% (Bun Lam Khao and Jingouzi), these differences in fracture rates are not statistically different between these assemblages (χ^2^ 6.613, p = 0.251, note Khok Phamon Di excluded due to zero observations).

**Table 4:** Prevalence of limb bone fractures by element (sides combined) in Southeast and East Asian archaeological assemblages.

Assemblage	Clavicle	Humerus	Radius	Ulna	Femur	Tibia	Fibula	Total (excluding clavicles)	Date (years BP)
Con Co Ngua^1^ 1979	n/a	2/45 (4.4%)	0/27 (0.0%)	0/21 (0.0%)	3/46 (6.5%)	0/29 (0.0%)	0/12 (0.0%)	5/180 (2.8%)	6700–6200
Con Co Ngua 2013	3/69 (4.3%)	0/102 (0.0%)	5/89 (5.6%)	3/110 (2.7%)	1/95 (1.1%)	1/60 (1.7%)	1/62 (1.6%)	11/518 (2.1%)	"
**Con Co Ngua** **Total**	**3/69 (4.3%)**	**2/147 (1.4%)**	**5/116 (4.3%)**	**3/131 (2.3%)**	**4/141 (2.8%)**	**1/89 (1.1%)**	**1/74 (1.4)**	**16/698** **(2.3%)**	"
Vietnam Metal period sites^1^	n/a	0/23^h^ (0.0%)	1/14^r^ (7.1%)	1/14^u^ (7.1%)	0/25^f^ (0.0%)	0/18^t^ (0.0%)	0/11^fi^ (0.0%)	2/105 (1.9%)	3300–1700
Khok Phanom Di^2^ Thailand	2/106 (1.9%)	0/104 (0.0%)	0/102 (0.0%)	0/74 (0.0%)	0/85 (0.0%)	0/90 (0.0%)	0/48 (0.0%)	0/503 (0.0%)	3950–3450
Ban Lum Khao ^2^ Thailand	1/39 (2.6%)	0/37 (0.0%)	3/48 (6.3%)	4/40 (10.0%)	0/37 (0.0%)	0/34 (0.0%)	1/29 (3.4%)	8/225 (3.5%)	3350–2450
Non Ban Jak^3^ Thailand	1/69 (1.4%)	0/77 (0.0%)	4/57 (7.0%)	1/60 (1.7%)	0/65 (0.0%)	0/66 (0.0%)	0/62 (0.0%)	6/387 (1.6%)	1650–1200
Jinggouzi^4^ Inner Mongolia	n/a	1/32 (3.1%)	0/33 (0.0%)	3/32 (9.3%)	2/48 (4.2%)	2/43 (4.7%)	0/37 (0.0%)	8/225 (3.5%)	2950–2550
Lamadong^4^ Northeast China	n/a	2/231 (0.9%)	6/151 (4.0%)	1/158 (0.6%)	2/316 (0.6%)	6/307 (2.0%)	5/161 (3.1%)	22/1324 (1.7%)	1730–1511

^1^[6]

^2^[4]

^3^[7]

^4^[55]

When examining the frequency of upper (excluding clavicles) and lower limb fracture rates, there are no statistically significant differences in any of the assemblages examined (Table 5). However, the prevailing pattern for the assemblages from Neolithic through to Iron Age Thailand and Metal Period Vietnam is for higher rates of upper body fractures. Jingouzi (an early pastoral site in Mongolia) is unique in displaying higher lower body fracture rates, while Con Co Ngua (combined 1979/80 and 2013 series) is similar to the agropastoral site of Lamadong in having similar upper and lower body fracture rates.

**Table 5:** Comparison of upper and lower limbs fractures for Southeast and East Asian archaeological assemblages.

Assemblage	Upper limbs	Lower limbs	Significance
Con Co Ngua Total	10/94 (2.5%)	6/304 (2.0%)	χ2 = 0.244, p = 0.621
Vietnam Metal period sites	2/52 (3.9%)	0/54 (0.0%)	N/A
Khok Phanom Di	0/280 (0.0%)	0/223 (0.0%)	N/A
Ban Lum Khao	7/122 (5.7%)	1/100 (1.0%)	FET = 0.059
Non Ban Jak	5/194 (2.6%)	1/193 (0.5%)	FET = 0.109
Jinggouzi	1/97 (1.0%)	4/128 (3.1%)	FET = 0.283
Lamadong	13/784 (1.7%)	22/1324 (1.7%)	χ2 = 0.000, p = 0.991

Data and sources derived from Table 4

## Discussion

It is argued that the hunter-gatherers buried at Con Co Ngua lived during a period of increased wild animal herd management, or increased hunting and consumption of water buffalo, over two millennia prior to evidence of domestication. The comparison of this early site in northern Vietnam to broader regional sites with distinct subsistence bases is an opportunity to test the risk of limb bone trauma in (potentially) sedentary hunter-gatherers compared with pastoralists and rice agriculturalists. Risk appears to have declined in tandem with the establishment and intensification of agriculture, although not significantly with a decrease from 2.3% of limb bone fractures at CCN during the Da But period to 1.9% at Metal period sites. The latter prevalence is similar to the fully agricultural site of Non Ban Jak, which utilised domesticated cattle and water buffalo. However, results are variable and reflect the wide temporal, cultural, and environmental contexts of these populations. Excluding other elements, the *distribution* of the limb bone fractures between sites could be more revealing. For the most part, it was the larger limb bones (humerus, femur, tibia) that were fractured in both the Chinese pastoral and agropastoral populations, and at Con Co Ngua. Potentially, horseback riding could be implicated as one cause of some of the fractures at Lamadong and Jinggouzi, in addition to interaction with other animal domesticates, particularly at Jinggouzi where 89 percent of tombs contained animal remains [55]. Interactions with large animals at these sites may have exposed individuals to greater danger of injuring these larger elements, which take greater force to fracture.

The majority of the injuries of those buried at CCN are not suggestive of interpersonal violence. There is just one case of a possible parry fracture to the right ulna (see Table 2, M17a), but the fact that this male also sustained fractures to the left arm and right leg suggests serious but accidental trauma. It is probable most injuries observed at CCN were accidental, and the nature of the accidents can be explored further. For example, one individual (M2a) had a Colles’ fracture of the distal radius suggestive of a fall. These are common in a modern clinical context with the mechanism of injury linked to accidental falls on level ground, falls following a push, or in the unsteady elderly [78, 79]. For instance, in a study of hospital admissions in Denmark over one year 87% (344/394) of women and 64% (63/99) of men admitted with distal radius fractures had fallen with the majority being Colles’ type [79].

Our hypothesis that males and females will display different patterns and/or rates of trauma due to putative division of labour differences were somewhat supported. The clinical literature indicates males are more commonly injured by cows and bulls, typically in an agricultural environment, than females (Bush et al., 1986; Dogan et al., 2008; Murphy et al., 2010; Norwood et al., 2000). It has been suggested through the analysis of limb bone muscle entheses that differences in activity patterns existed between males and females buried at CCN [80]. The pattern of fractures to the axial skeleton in CCN males suffering vertebrae and pelvic fractures, and fractures of large limb bones, highlight potentially important differences in the causes of trauma, some of which could be due to physical exchanges with wild animals and sexual divisions in labour. However, while males displayed both upper and lower body trauma, and females displayed upper body trauma for the most part, the differences were not statistically significant.

It is difficult to determine the specific events that caused injuries in the past as different events can cause similar injuries. Many of the fractures (including those of the clavicle and forearms) at CCN could easily be attributed to falls (or other accidents) or to encounters with wild animals (see especially [81]). It is likely that in some instances animals were responsible for fractures or other trauma, but to differentiate these from other behaviours is problematic. Comparing the types and patterns of trauma from other mechanisms, such as falls, with those sustained by bovine in the contemporary literature could help tease out some of these differences. Little research has been conducted attempting to identify the patterns of trauma experienced during interactions with wild animals, behaviours that presumably commonly occurred prior to animal domestication. The clinical literature provides a tentative basis for the patterns of trauma expected from bovine and accidental injuries sustained during the management of, and interaction with, large animals. These come with caveats as reports relate predominantly to modern milking and farming practices. There are, however, injuries that appear indicative of bovine interactions when only injuries from bulls, bison, and documented water buffalo attacks are considered (see [19, 22] for example). These include cervical vertebral fractures, and fractures of the ribs, pelvis, and upper and lower limbs, specifically the humerus, femur, and tibia: all elements of which displayed healed trauma among CCN males.

### Accidental falls versus bovine induced injuries

Post-cranial fractures from falls commonly include the sternum and cervical vertebrae (from head and neck hyperflexion with the chin striking the sternum), ribs, and femora [82–84]. Other elements fractured to a lesser degree include clavicles, limb bones, vertebrae (thoracic and lumbar), and the pelvis [82–86]. In the upper limb, fractures from falls tend to be localised to the elbow and distal forearm while in the lower limb the proximal femur, distal tibia, fibula, and ankle are affected [83]. The severity and distribution of injuries increase with height, type of impact surface, velocity, and the position of the body upon impact [83, 87].

**Fig 6:**
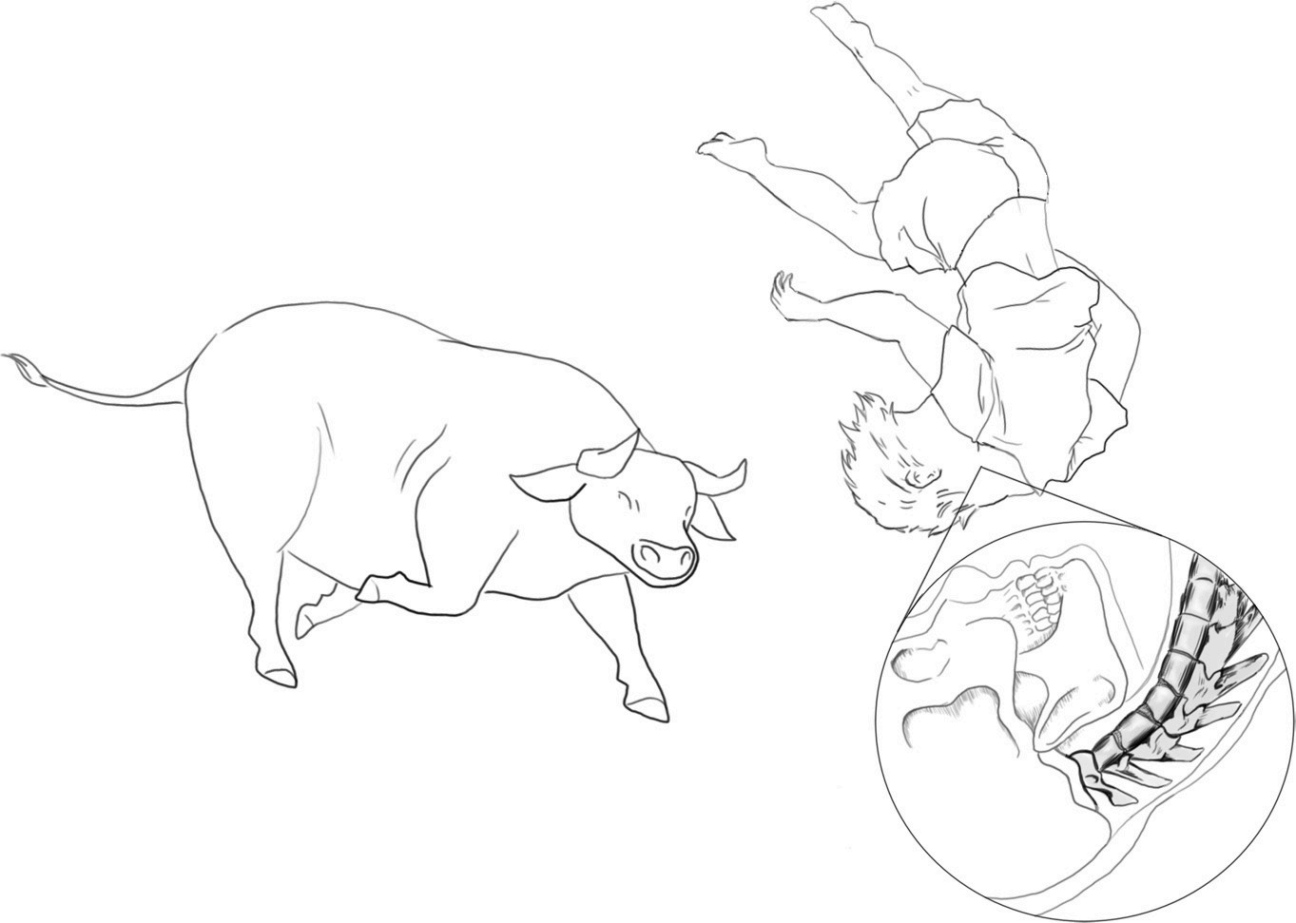
An example of hyperflexion of the neck following being tossed into the air. Image credit Rebecca Benham.

Accidental fall injuries are caused by sudden vertical deceleration and transfer of energy through the body [84], which is also a factor in bovine trauma (in addition to horizontal and deceleration injuries). In one comtemporary case an individual sustained rib, forearm, scapula, and dental alveolar fractures in addition to soft tissue trauma after being knocked to the ground at least seven times by a dairy bull [88]. Water buffalo can weigh up to 600 kg and stand 2 m tall [89] and have been known to toss an adult male several meters into the air leading to head or neck trauma [90]. Hyperflexion of the neck causes cervical vertebrae fractures and dislocation (Fig 6), comparable to falls of less than 3 metres that have been shown to cause axial fractures [91]. Though, bovine create greater velocity during an incident. This is consistent with trauma observed in one CCN male (M127) with cervical vertebra trauma. Similarly, one could be injured from being knocked down or manoeuvring any large animal.

Many of the injuries caused by bovids are comparable in severity to high-energy injuries from other scenarios such as vehicular (both pedestrian and intra-vehicular) incidents [92, 93]. Unsurprisingly, fractures are one of the most frequent types of trauma sustained during these bovid interactions [21–25, 94]. Other injuries caused by bovids include dislocations, lacerations and punctures, organ perforation, and soft tissue damage with multiple trauma in different regions of the body being common [23, 94].

Documented patterns of trauma of individuals working with domesticated livestock may assist in the interpretation of antemortem injuries found at CCN. In a farming context, Murphy et al. [24] found that 17% of individuals with cow-related fractures had suffered an open fracture, involving the tibiae, the forearm, metatarsals, wrist, and fingers. At CCN, one male had a comminuted tibial fracture, which had formed a cloaca indicating secondary infection that could have formed from an open fracture. Of lower limb injuries, tibial plateau fractures caused by cows in this modern farming context were found to predominate (hospital admissions in Ireland over 10 years, 5/27 fractures, 18.5%) [24]. Plateau fractures only account for approximately 1% of all modern fractures, making their incidence sustained from cows in the Irish study high [73]. Similarly, femoral trochanteric fractures are rare [73] but were found in four individuals (3.1%) in a study of cow and live-stock trauma in Iran over 5 years (n = 129 injured) probably caused by a blow to, or, landing on the hip.

On considering the literature of bovid attacks outside of a farming context (water buffalo and bison), injuries include rib, pelvis, cervical vertebrae, and skull fractures, and punctures and lacerations to the face, chest, abdomen, buttocks, pelvis, legs, and extremities caused by goring, charging, head-butting, trampling, and being crushed and tossed [19, 20, 22, 23]. Buffalo can strike multiple times repeatedly butting and trampling their victim [92, 93, 95]. Individuals attacked by bovids tend to sustain more than one injury [88]. Unfortunately, clinical studies of bovine related trauma often do not provide information regarding the specific skeletal element, exact location, or fracture type. Because being knocked down and falling are causes of trauma in falls and in bovine encounters similar injuries including distal radius fractures can occur. The skull is less frequently injured by bovine compared to accidental falls. Furthermore, only a quarter of those injured by bovines may suffer fractured bones [21, 23, 25] with those suffering soft tissue trauma not usually visible in the archaeological record.

In summary, a review of the clinical literature indicates similarities in the skeletal elements commonly fractured in falls and bovid interactions (cervical vertebrae, ribs, clavicles, pelvis, and forearm). Falls (as opposed to direct bovid induced trauma), however, are more often associated with skeletal fractures to the skull, sternum, thoracic and lumbar vertebrae, elbow, and distal tibia and fibula. Clinically, fractures caused by bovids involve multiple trauma, open fractures, cervical vertebrae, larger limb bones, the tibial plateau, scapula, trochanteric fractures, long bones of the hands and feet, and skull.

The skeletal fractures CCN individuals experienced fall into three categories:

1. indirect trauma that was more likely to have been accidental (for example an oblique fracture of the radial diaphysis),

2. trauma that could have been caused either accidentally or from a direct blow (for example a fracture of the radial styloid process can be due to a direct blow to the wrist or by tension placed on the radiocarpal ligament),

3. and fractures that could have been caused either by a direct force, falling onto an object, or by another person or animal (for example a transverse fracture of the ulna and femur, and amputation of metatarsals four and five).

Excluding injuries more likely to have been caused accidentally, two adult males suffered injuries that have the potential to be related to other behaviours, including interacting with animals. Burial M127a had a fractured clavicle, which in addition to a fall onto an outstretched arm can occur from a direct or lateral blow to the shoulder. In addition, he fractured a cervical vertebra from hyperflexion and compression of the neck. Similarly, the transverse fracture of the femur and fractured left pubic ramus of Burial M126a are clinically usually associated bovine, equestrian accidents, and crushing injuries in addition to falls. No individuals sustained tibial plateau, trochanteric, or scapula fractures. Clinically, however, the former two are found in farming contexts, such as managing cattle as opposed to non-domesticated animals. The pattern of trauma, more so than specific injuries, is perhaps a more useful gauge of the skeletal injuries that could have been caused by large animal encounters compared with other accidental scenarios. Nevertheless, it is acknowledged that in the absence of direct evidence (an imbedded arrow head for example) it is not often possible to determine the specific cause of injury from skeletal remains alone. Moreover, it is impossible to determine if multiple injuries occured in a single event or as repeat trauma over time. Injury recidivism and risk of fracture, including vertebral fractures, escalates following an initial injury and the risk ratio between men and women does not differ [96, 97].

We cannot definitively say that adults buried at Con Co Ngua were injured by wild water buffalo as other circumstances or interactions with wildlife present in the local environment could have fractured and injured limbs and extremities [98–100]. However, based on the dominance of bovid remains with evidence of butchering at CCN, it is probable that some form of wild herd management or specific hunting strategy (for instance, trapping) contributed to the trauma observed in this skeletal assemblage. The injuries individuals suffered in life serve as a permanent record of the risks they were exposed to, and the sheer quantity of large bovid remains recovered from CCN implies they interacted with these wild beasts on a regular basis.

It is clear that more research is necessary to explore the frequency and types of physical injuries at sites globally during periods of large wild animal interaction and/or domestication. The primary aim of this paper was to determine if there was evidence for traumatic injuries indicative of animal interactions in a settlement that has extensive evidence for increasing contact with bovids prior to domestication in the region. We suggest that the cultural context and overall pattern of multiple traumas, including axial injuries specifically to the cervical vertebrae, pelvis, and largest limb bones could be useful for distinguishing large wild animal interactions from other causes of trauma in the past and these should be considered in future studies.

It has been hypothesised that hunter-gatherer lifestyles predispose individuals to greater risks of injury and higher fracture frequencies compared to established agriculturalists [101] but this ignores the process of wild animal domestication, management, and associated risks that may have occurred simultaneously, or just prior to, the advent of agriculture. By the time evidence of domestication is visible in the archaeological record it may have been long established. Potentially, the precursor to domestication included managing wild animals and herds, putting hunter-gatherer groups at great personal risk as they interacted more intimately with these large unpredictable animals, many of which, like the water buffalo in Vietnam, are now established and entrenched in tradition.
